# Taking Stock of Knowledge Transfer Studies: Finding Ways Forward

**DOI:** 10.1007/s00267-023-01877-y

**Published:** 2023-09-09

**Authors:** Carina Lundmark, Jens Nilsson, Anna Krook-Riekkola

**Affiliations:** 1 Department of Social Sciences, Technology and Arts, Luleå University of Technology, SE-971 87 Luleå, Sweden; 2 Department of Engineering Sciences and Mathematics, Luleå University of Technology, SE-971 87 Luleå, Sweden

## Abstract

Knowledge transfer (KT) from academia to practice is important in many fields, but comprehensive studies on identifying the most effective forms of KT are scarce. This paper aims to provide an overview of KT theory and presents a cross-disciplinary scoping review of empirically oriented peer-reviewed articles. The review offers guidance for researchers seeking to communicate effectively with practitioners. It explores the effects of research communications, delves into the understanding and measurement of these effects, attempts to identify the most effective forms of communication, and highlights important considerations when designing KT strategies. Few studies in our sample (eight of 27) systematically measured effects of KT, and merely four studies compared multiple forms of KT. Nevertheless, most studies estimated effects from KT, regardless of the chosen form (e.g., workshops or lectures). Most studies estimated knowledge change as the primary outcome. Additionally, several studies explored altered beliefs such as increased self-efficacy. A third of the studies addressed how the knowledge was applied, ranging from sharing information to developing new habits. The identified effects were, however, both small and volatile. Our findings underscore the significance of continuity and repeated interactions to enhance the impact of KT initiatives. Furthermore, researchers need to develop a comprehensive set of tools to facilitate successful KT, considering factors such as expertise, communication skills, trust-building, and participant-centered approaches. By employing these strategies, researchers can effectively bridge the gap between academia and practice, facilitating successful KT in various fields.

## Introduction

Previous research in natural resource management (NRM) shows that scientific knowledge with strong potential to have an impact tends to stay within academia and offer little use to those who struggle with ongoing management problems. This long-observed problem encompasses diverse research areas such as ecosystem management (Roux et al. 2006), landscape ecology (Perera et al. 2006), freshwater ecology (Cullen et al. 2001), wildland fire management (Adams et al. 2017), water management (Hattermann et al. 2011), and marine management (Klütsch and Laikre 2021; Sandström et al. 2019). Using conservation genetics as an example, genetic diversity tends to be disregarded in ongoing management even though it is scientifically acknowledged as a key element of biodiversity and highly important for species’ adaptation to changes in their environment (Taylor et al. 2017). The effects of not including these research results in practice can be detrimental and may increase due to climate change (Harrisson et al. 2014). For instance, Cvitanovic et al. (2014) showed that managers of Australian marine protected areas need improved understanding of adaptation as a strategy to face the risks imposed by climate change. Taylor et al. (2017, p. 231) found that conservation practitioners have much to gain from collaborating with conservation genetics researchers but “do not necessarily know how to talk to [the researchers] or fully understand how genetics might benefit them”. Researchers also face many barriers when attempting to interact with practitioners. As early as 2001, Cullen et al. (p. 3) noted that “research groups commonly have limited skills and resources to carry out this task, and it is not part of the normal reward structures of science”.

Despite these barriers, there have been numerous attempts to close the knowledge gap between research and practice in the NRM field. To continue the above example from conservation genetics, Stetz et al. (2011) developed an online resource to assist managers with limited genetics experience in their efforts to monitor and assess population developments. In addition to inquiring into the potential of genetic tools for conservation practitioners, Hoban et al. (2013) recommended closer collaborations (e.g., partnerships) and increased efforts in conveying research applications. Sandström et al. (2019) used lectures and deliberative group discussions to improve the knowledge on genetic diversity among managers responsible for marine protected areas in the Baltic Sea. They found that lectures were most effective in increasing managers’ knowledge but recommended repeated interaction, as the effects from knowledge transfer (KT) disappeared over a few months.

It is not possible to draw general conclusions about what approach works best from case studies such as these. Literature reviews can provide a more general picture, but they are scarce in the NRM field and tend to address KT as a pathway for future action, rather than as the prime focus of research (Razak et al. 2022; Wennerström et al. 2017). Studies targeting KT also tend to have a relatively restricted focus. For instance, Posner and Cvitanovic (2019) compared pros and cons of different techniques that can be used for measuring the effect of boundary-spanning activities between science and practice (e.g., through network analysis, interviews, and surveys). They also recommended measuring effects from KT as a theme for further research.

In response to the limitations highlighted in previous studies, the aim of this paper is to provide an overview of KT in practical application. We will conduct a cross-disciplinary literature review of empirical research of KT processes that encompasses communication between scientists and practitioners. To address knowledge gaps identified in previous research, our emphasis lies on endeavors to measure effects from researchers’ communication. Our research questions are: *What effects emerge from research communications? How are effects understood and measured? Which forms of communications are most efficient? What is important to consider when designing processes of knowledge transfer?*

## Theoretical Overview

Knowledge transfer is commonly defined as a *communicative process* that involves one or more knowledge providers/senders and knowledge receivers, though their roles can alternate during the process (Kuiken and van der Sijde 2011; Szulanski 1996). In the NRM literature, the knowledge provider is commonly a researcher, whereas the receivers are often individuals who work outside academia, mainly public officials/managers but also NGOs or the public (Johansson et al. 2019; Klütsch and Laikre 2021; Perera et al. 2006; Sandström et al. 2019). In business research, the focus is on KT between universities and industry (Kuiken and van der Sijde 2011), within firms (Spraggon and Bodolica 2012), or between firms (Milagres and Burcharth 2019). The multi-directionality of KT is particularly pronounced in studies on co-production of knowledge (Roux et al. 2006; Tranfield et al. 2004).

Knowledge is expected to be absorbed or learned through the KT process. Additionally, knowledge is expected to be put to use—that is, to result in some form of action (De Luca and Rubio 2019; Gera 2012; Kuiken and van der Sijde 2011). The content of the knowledge in a KT process clearly varies significantly. Apart from the subject matter itself, it can also vary in terms of complexity (Cullen et al. 2001), quality and quantity of the information (De Luca and Rubio 2019), context dependency (Milagres and Burcharth 2019), and whether it is explicit or tacit (Gera 2012; Roux et al. 2006) and independent or systemic in character (Milagres and Burcharth 2019). From the perspective of the receiver, the novelty and appropriateness of the communicated message can also vary (De Luca and Rubio 2019; Milagres and Burcharth 2019; Spraggon and Bodolica 2012).

Spraggon and Bodolica (2012) argue that the selected form of KT should match the characteristics of the knowledge itself. To facilitate this, they developed a taxonomy of KT processes with four dimensions. The first dimension, degree of programmability, refers to the extent to which the process is more planned or spontaneous. The second dimension, level of discretion, ranges from freedom to act upon a KT process to an obligation to do so. The third dimension, scope of coverage, ranges from broad to narrow and refers to the number of participants involved. The final dimension, process orientation, refers to the medium for communication, ranging from technology-based to people-based. When these dimensions are combined, four ideal types emerge: static virtual processes, dynamic virtual processes, canonical face-to-face processes, and non-canonical face-to-face processes. *Static virtual processes* encompass, for instance, unaddressed documents of various kinds, such as reports and databases. They commonly have broad coverage and are easy to access but tend not to be adjusted to recipients’ needs. *Dynamic virtual processes* also rely on technology and are broad in scope but enable knowledge exchange (e.g., through intranets, blogs, telephone, and email). Turning to narrow scope of coverage, we find that teaching is an example of *canonical face-to-face processes* that are planned, focused on people, and have low level of discretion (Spraggon and Bodolica 2012). Finally, *non-canonical face-to-face processes* are spontaneous and informal in character with a high level of discretion, such as when casually sharing information with a colleague. Based on this taxonomy, Spraggon and Bodolica (2012 p. 1280) present propositions that can guide KT. For instance, “[v]irtual processes are particularly suitable for the attainment of knowledge outcomes that are more general, impersonal, acontextual and atemporal”. Richer media, such as face-to-face communication, are recommended when there is a need for personalization and direct feedback. While Spraggon and Bodolica’s focus is on intra-firm KT, we argue that their theoretical contributions are applicable across disciplinary boundaries. With their work dating back to 2012, we note that virtual (online) processes for KT are considerably more prominent today, particularly after the COVID-19 pandemic (cf. Agrifoglio et al. 2022; Fehl et al. 2022; Saide and Sheng 2021).

A basic assumption in KT research is that (new) knowledge is adopted and assimilated into the receivers’ existing knowledge structure. In the literature on KT, this step is often not explicated. Roux et al. (2006) and Gera (2012) are exceptions, distinguishing between different ways that knowledge assimilation may happen, such as tacit knowledge sharing and externalization (i.e., when tacit knowledge is made explicit). Gera (2012 p. 253) argues that internalization “is a process of learning about adequate replication of specific tasks”. We choose a broader interpretation, encompassing not only replication but also adaptation of the knowledge to a specific situation or problem solving.

This broader interpretation aligns with recent studies emphasizing context-sensitive KT designs, adapted to the practitioners’ professional work. For example, Belanger-Gravel et al. (2023) studied health practitioners' use of behavioral science related knowledge in health promotion interventions. Their results showed a difficulty to fully integrate the behavioral science framework and theories, primarily due to prevailing knowledge structures and lack of contextual adaptation. More specifically, the practitioners perceived that the health interventions did not fully match their profession, their knowledge base and available resources. Tedim et al. (2021) used a context-sensitive and problem-solving approach in the context of wildfire suppression. They involved practitioners through-out the KT process and worked transdisciplinary to explore both experiential and traditional knowledge.

Previous research highlights factors that can have an impact on KT processes (enablers or disablers). Some focus on the individual level while others focus on organizations, mainly firms (Milagres and Burcharth 2019; Szulanski 1996). As we are interested in various ways of exchanging knowledge with practitioners, such as public managers, we will focus on enablers that are individually oriented. Individually oriented factors largely fall into three categories of characteristics that are associated with a) *the actors involved*, b) *the content of the knowledge*, and c) *the staging of the process*. Actor-oriented characteristics primarily concern the receiving end of the KT and involve motivational factors ranging from culture and various beliefs (intrinsic motivation; Cullen et al. 2001; Roux et al. 2006; Spraggon and Bodolica 2012) to financial gains and career options (extrinsic motivation; Milagres and Burcharth 2019; Perkmann et al. 2013). Actor-oriented factors also involve cognitive features such as capacity to understand/absorptive capacity (Milagres and Burcharth 2019; Roux et al. 2006; Szulanski 1996), as well as practically oriented features such as time available to engage in the KT process (Cullen et al. 2001; Spraggon and Bodolica 2012). Turning to the knowledge producer/sender, previous studies mainly highlight disseminative capacity, which can involve pedagogical skills and capacity to foster commitment among the participants (Kuiken and van der Sijde 2011).

Barriers and enablers relating to the content of the knowledge and the staging of the process are commonly practically oriented. Spraggon and Bodolica (2012) elaborate on the complexity and difficulty of the knowledge to be transmitted, while Szulanski (1996) also addresses the reliability of the source (knowledge producer/sender/provider) and is strongly linked to the varying traits of the actors who are expected to use the knowledge, such as prior knowledge and understanding (Cullen et al. 2001). As people’s learning strategies tend to vary, Cullen et al. (2001) recommend the use of different forms for knowledge communication. They also highlight the importance of ensuring that the communicated message is relevant and applicable.

From the theoretical overview presented thus far, we have already identified some tentative answers to our research question regarding *what, according to previous research, is important to consider when designing KT*. However, the practical recommendations are sparse and offer general rather than specific guidance on how to bridge the science–practice gap. Notably, theoretically oriented research rarely specifies the outcome of KT. Rajaeian et al. (2018 p. 40) note that “the effectiveness of knowledge transfer activities is commonly neglected” (see also Milagres and Burcharth 2019; Perkmann et al. 2013). There are exceptions among the scholars referred to thus far, such as Szulanski (1996) and Milagres and Burcharth (2019), but they focus on effects of KT within or between firms, such as innovation. In this literature review, we will turn to several disciplines to identify which forms of KT have been identified as the most effective.

## Methods

Scoping reviews, rather than a systematic literature review, are recommended in complex research areas (Arksey and O’Malley 2005). A scoping review is also recommended when focusing on key concepts and using a theoretical framework for collecting and summarizing the results. Colquhoun et al. (2014 pp. 1292 and 1294) define a scoping review as addressing “explanatory research questions aimed at mapping key concepts, types of evidence, and gaps in research related to a defined area or field by systematically searching, selecting, and synthesizing existing knowledge”. In our case, the scoping review is motivated by our focus on key concepts and the application of Spraggon and Bodolica’s (2012) taxonomy. Additionally, we address acknowledge gaps in a highly complex research area. We applied a scoping review following the five stages developed by Arksey and O’Malley (2005); 1) identification of research questions, 2) identifying relevant studies, 3) study selection, 4) data charting, and 5) data analysis. The sixth step, stakeholder consultation, is optional and intended to provide feedback on study design and usefulness. As our main target group is researchers in the NRM field, we collected feedback through scientific seminars involving scientists with such experience.

Initially, the concept of KT was defined, which led to the identification of relevant search words. A librarian was consulted to create the search strings with Boolean operators to ensure the identification of relevant studies. The final search string was as follows:

("knowledge transfer*" OR "knowledge diffusi*" OR "knowledge dissemin*" OR "knowledge communicat*" OR “knowledge exchange") AND (manager* OR “public official*” OR bureaucrat* OR "civil servant*" OR practitioner*) AND (effect* OR outcome* OR impact*)

Several search strings were tested prior to this selection. We elaborated upon different ways to capture practitioners. We decided on broad terms to enable relevance across disciplines, excluding specific occupational terms such as teacher, principal, and superintendent. We also settled for variations on different words for knowledge transfer rather than specific forms of communication (e.g., presentations, lectures, seminars, etc.). When testing different search strings, we noted that most of the articles relevant to our study were published in recent years. We therefore decided to delimit our search to the years 2015–2020.

The academic databases used for the literature search were SCOPUS and Web of Science, as they provide reliable and broad coverage of abstracts from peer-reviewed published articles from a wide range of disciplines in the science, technology, social sciences, humanities, and arts fields. The search resulted in 857 hits when using the Web of Science database and 905 hits using Scopus, with English language as a filter and 2015–2020 as the selected year range.

**Fig. 1:**
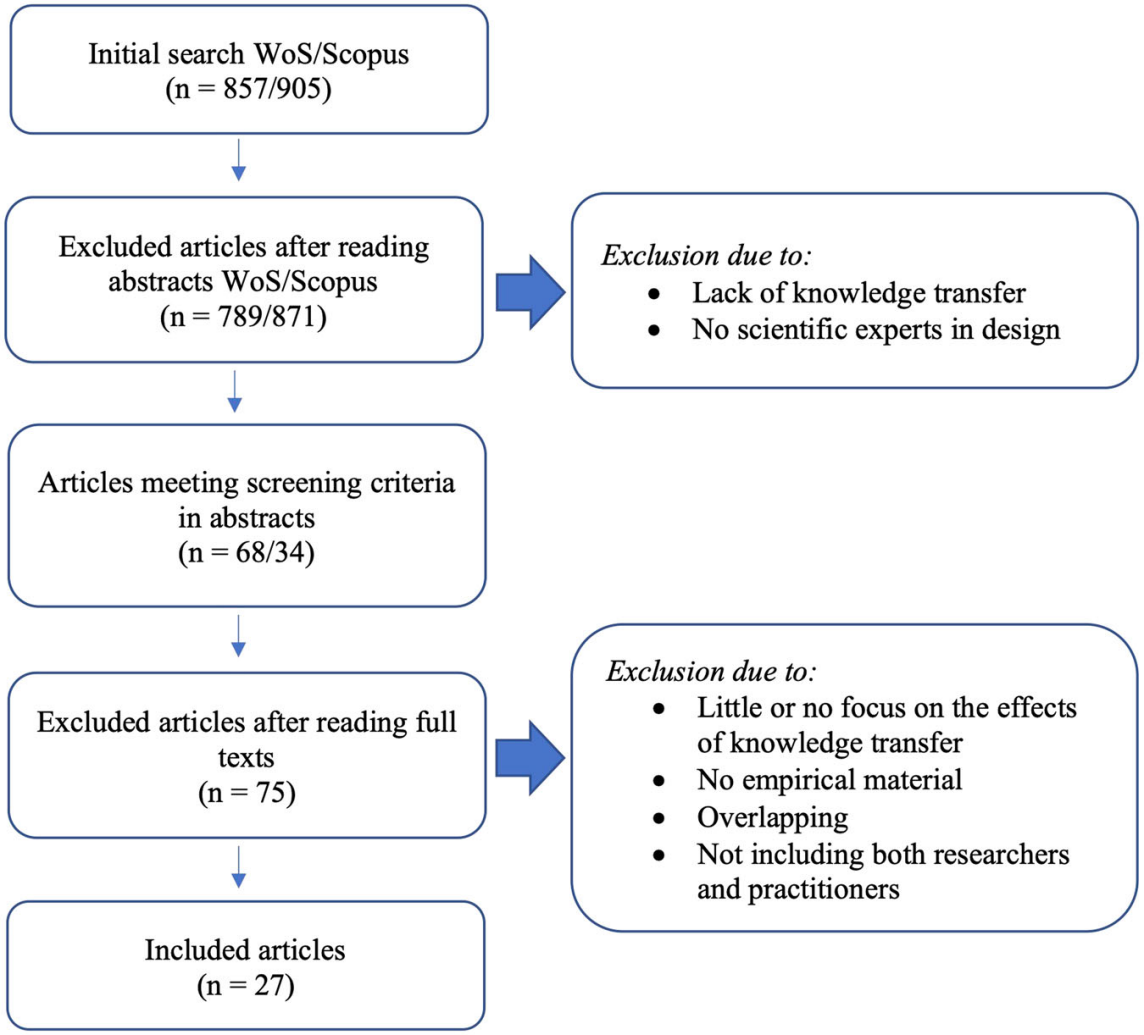
Flow chart for the screening process

We limited our search to articles focused on a) empirical studies of KT processes, b) KT processes involving scientists *and* practitioners, and c) attempts to measure the effect of KT. Our justification for choosing empirical studies was to enable in-depth analysis of the applied forms of KT. A flow chart for the process of narrowing the pool and screening criteria is presented in Fig. 1. The abstracts were read to determine the relevance of the findings. Gray literature was excluded, as well as book chapters and conference abstracts. When reviewing the abstracts, 102 articles met our predefined requirements (68 from WoS, 34 from Scopus) and qualified for a more detailed data analysis. Forty-five of these were excluded due to little or no focus on the effects of KT, or little or no empirical material, or overlapping. We also excluded articles that did not include both researchers and practitioners. For instance, studies exploring the effects of different forms of education in a classroom situation with students were excluded. One of the authors reviewed the findings from the Web of Science, while another author reviewed the findings from Scopus. All articles that were identified as potentially relevant were reviewed by two of the authors. A total of 75 articles were excluded in this part of the process. Consequently, 27 articles were identified for inclusion in the qualitative content analysis of the entire texts, which constituted the final step of our literature review. One article was published in 2021 but was available for early access in 2020, making it eligible for our search.

### Data Charting and Analysis

A targeted qualitative content analysis was pursued following Hsieh and Shannon (2005; see also Mayring 2014). The analytical scheme encompassed six categories: a) the target group(s), b) the character of the KT, c) methodology, d) main findings (reported effects from KT), and e) lessons for further studies. A final category (f) included basic background information (area of study, location of the study/study sites, and year of data collection). Starting with the target group, we noted the receivers of the information—that is, the specific types of practitioners addressed. We used the dimensions developed by Spraggon and Bodolica (2012) to categorize the data on different forms of KT; degree of programmability, level of discretion, scope of coverage, and process orientation, also noting the specific forms of KT used (e.g., presentations, documents, or informal networks). The analysis of full texts also included notes on methodology (e.g., whether effects from KT were explored through interviews or surveys). We also investigated whether the effects were measured in a systematic way and whether effects measurements were extended over time. The main results included identified effects from KT (e.g., increased knowledge or action). The final category, lessons for further studies, was reserved for suggestions or recommendations on KT design and implementation identified in the previous studies. The main findings for each category were condensed and presented in spreadsheet tables. From these condensed texts and tables, conclusions were drawn to fulfill the aim of the article.

## Findings

The 27 publications identified through our literature review are listed in Table 1 (see References for details), along with the following background information: scientific field or area of study, location of study, and methods. Multiple fields of research were represented, from information systems management to healthcare, as well as our initial focus on NRM research. Healthcare was the most common field of research, applicable to nine of the studies. Almost half of the studies explored KT in a European setting. Methods of the investigations showed great diversity, involving interviews, focus groups, and various forms of participatory observations, although surveys and interviews were most frequently used. This result corresponds with the results in the literature review by Campbell and Moore (2018) included in our sample.

**Table 1:** Overview of selected articles on knowledge transfer

Publication	Area of study	Location of study	Methods
Aelbrecht and Stevens (2015)	Urban design	The United Kingdom	Ethnographic
Andrews et al. (2020)	Health and social care	Scotland and Wales	Focus groups, interviews
Annemans and Heylighen (2021)	Building design	Belgium	Content analysis
Arevalo et al. (2020)	Water management	The Netherlands	Focus groups, surveys
Bruce and O’Callaghan (2016)	Knowledge brokering	The United Kingdom	Workshop, interviews
Campbell and Moore (2018)	Health policy and practice	International	Varying (e.g., workshops, online forums, masterclasses, policy briefs, partnerships)
Dowson (2019)	Medical education	England	Surveys, interviews, transcripts from telephone coaching
Gervais et al. (2016)	Knowledge transfer	International	Varying
Hains et al. (2019)	Medicine	Australia	Survey
Hakkarainen et al. (2020)	Fisheries management	Zanzibar	Interviews, participant observation
Hall et al. (2019)	Library and information services	The United Kingdom	Survey and focus groups
Hambidge et al. (2019)	Higher education	The United Kingdom (with international participation)	Mixed methods
Hurley et al. (2017)	Urban planning	Australia	Direct observations, survey
Janousek and Blair (2018)	Local government management	The United States	Focus groups
Kremer et al. (2017)	Public health	The Republic of Fiji	Survey, interviews
Lee et al. (2018)	Medicine	Canada	Surveys
Lundmark et al. (2019)	Marine management	Sweden	Surveys
Lundmark et al. (2017)	Marine management	Sweden	Surveys
McCart et al. (2020)	Education and rehabilitation	The United States	Surveys
Oronje et al. (2019)	Health policy	Kenya and Malawi	Surveys, interviews
Packer et al. (2019)	Soil and land management	Australia	Surveys
Porterfield et al. (2017)	Public health	The United States	Survey, interviews
Rajaeian et al. (2018)	Information systems management	International	Interviews, survey
Sass et al. (2019)	Primary care	The United Kingdom	Focus group, interviews, surveys
Ugolini et al. (2015)	Forestry	Italy	Survey
Winarto et al. (2017)	Farming	Indonesia	Direct observations, focus groups
Wright and Wright (2017)	Art psychotherapy	The United Kingdom	Participatory observations

See References for full details on the publications

Next, we dig deeper into what has been done empirically to capture KT from science to practice and explore what is important to consider when designing such communication processes. The results are summarized in Tables 2 and 3 and further explicated in three subsections covering the content and form of communication, investigated effects, and lessons learned.

**Table 2:** Overview of different forms of knowledge communication

Publication	Form of communication	Degree of programmability	Level of discretion	Scope of coverage	Process orientation
Aelbrecht and Stevens (2015)	Presentations and informal talks	High	High	Narrow	People-oriented
Andrews et al. (2020)	Storytelling and action-learning events	High	High	Narrow	People- & technology-oriented
Annemans and Heylighen (2021)	Workshops, presentations, and documents	Relatively high	High	Narrow	People-oriented
Arevalo et al. (2020)	Storytelling	High	High	Narrow	Technology-oriented
Bruce and O’Callaghan (2016)	Group discussions	Low	High	Narrow	People-oriented
Dowson (2019)	Action learning, telephone coaching	High	High	Relatively narrow	People- & technology-oriented
Hains et al. (2019)	Educational data program, personal meetings and group discussions	High	High	Broad	People- & technology-oriented
Hakkarainen et al. (2020)	Workshops	High	High	Narrow	People-oriented
Hall et al. (2019)	Workshops	High	High	Narrow	People-oriented
Hambidge et al. (2019)	Learning platform	Low	High	Narrow	People-oriented
Hurley et al. (2017)	Workshop	Low	High	Narrow	People-oriented
Janousek and Blair (2018)	Workshops, writing assistance	Relatively high	High	Narrow	People-oriented
Kremer et al. (2017)	Group discussion/deliberation	Relatively high	High	Narrow	People-oriented
Lee et al. (2018)	Workshops	High	High	Narrow	People-oriented
Lundmark et al. (2019)	Lectures, deliberative group discussions	High, relatively high	High	Narrow	People-oriented
Lundmark et al. (2017)	Lectures, deliberative group discussions	High, relatively high	High	Narrow	People-oriented
McCart et al. (2020)	Online training modules	High	High	Broad	Technology-oriented
Oronje et al. (2019)	Training, mentoring programs, discussion forums	High	High	Broad	People-oriented
Packer et al. (2019)	Workshops	Relatively high	High	Narrow	People-oriented
Porterfield et al. (2017)	Online communication platform	High	High	Broad	Technology-oriented
Rajaeian et al. (2018)	Collaborative research, informal communication, consultancy, presentations at seminars, publications.	Varying	Not relevant	Varying	People- & technology-oriented
Sass et al. (2019)	Educational program with practice-based learning	High	High	Narrow	People- & technology-oriented
Ugolini et al. (2015)	Publications, Internet, manuals, e-learning, technical meetings, seminars and conferences	Varying	Not relevant	Varying	People- & technology-oriented
Winarto et al. (2017)	Science field-shops	High	High	Not specified	People-oriented
Wright and Wright (2017)	Workshops, online resource hub	High	High	Narrow	People- & technology-oriented

Review articles are excluded from the table as their content in this regard is not possible to classify

### Content and Form of Communication

We recall from the theoretical overview that the content of the KT, besides the topic itself, can vary in terms of complexity (Cullen et al. 2001), quality and quantity of the information (De Luca and Rubio 2019). The studies in our sample highlight the need to adjust the content to the practitioners’ interests and needs. One of the most explicit examples is the literature review by Campbell and Moore (2018), outlining the results from 13 intervention studies on health policy and practice and concluding that messages should be tailored as different end-users prefer different content and format. Policymakers, for instance, preferred short summaries rather than detailed reports. On a similar note, staffers preferred story-focused policy briefs, while legislators preferred data-focused information.

Diverse forms of knowledge communications were used (Table 2), ranging from lectures and group discussions/workshops, which are relatively easy to organize and participate in, to collaborative research that is time-consuming for both researchers and practitioners. Most of the studies used a combination of communication types (e.g., presentations and informal talks, or workshops and online resources). All forms of communication in our sample are fact-/research-based. Two studies applied storytelling, thereby adding an element of emotion to the KT process to make the presentation interesting and engaging (Andrews et al. 2020; Arevalo et al. 2020). Table 2 also indicates large variation regarding the settings used, from personal interaction to online platforms. We have used Spraggon and Bodolica’s (2012) four-dimensional taxonomy to structure and outline these results further: degree of programmability (the extent to which the process is planned), level of discretion (freedom to act upon the knowledge in question), scope of coverage (number of participants), and process orientation (ranging from technology- to people-based).

Starting with *degree of programmability*, our results show that most KT efforts were planned, with a clear structure regarding what was communicated and how. Gervais et al. (2016) advocated careful planning, including targeting the communication to the respondents and designing measuring instruments. Research offered a common starting point for the studies in our sample, with the potential to benefit ongoing practice, and the communication was staged to transfer this knowledge in a way that was assumed to be well-suited to the target group(s). For instance, Andrews et al. (2020) used published research-based challenges on how to promote a better quality of life for older people using dialogic storytelling in communication with health practitioners and carers. Lundmark et al. (2017, 2019) presented the same knowledge content (genetic biodiversity in the context of the Baltic Sea) to managers using two different forms of KT that the managers rely on in their everyday work (lectures and group discussions). Sass et al. (2019) used a dementia-education program consisting of online learning material and practice-based learning to study alternations in knowledge, attitudes and practice among primary care staff. Only three KT processes in our sample were of a more spontaneous nature and had a different focus than the ones characterized by high programmability; Bruce and O’Callaghan (2016), Hambidge et al. (2019), and Hurley et al. (2017). Hambidge et al. (2019) was the only study in our sample explicitly aimed at stimulating *engagement* among the participants. Bruce and O’Callaghan (2016) aspired for improved knowledge, like most studies, but conducted KT research in a setting that diverged from the others, exploring effects from placement fellowships (i.e., researchers temporarily placed in a policy setting) to enable informal discussions and knowledge exchange. Both examples suggest that unconventional approaches to KT may call for more flexible and spontaneous designs. The third study (Hurley et al. 2017) aimed at improving research usability, which is an effect that cannot be classified into the three predominant categories (altered knowledge, altered beliefs and/or action). This also applies to the literature review by Campbell and Moore (2018) that explored increased use of research in population health policies and programs. Effects from KT will be further explored under a later heading.

The second dimension, *level of discretion*, was high in all studies in our sample when possible to classify (see Table 2), implying that the target actors experienced considerable freedom to act upon what they learned during the KT process. Commonly, the content of the KT was anticipated to be useful for the practitioners. For example, the KT explored by Dowson (2019) aimed to promote knowledge and confidence in clinical communication among healthcare professionals. Similarly, the KT studied by Hains et al. (2019) was intended to improve knowledge on how to treat severe asthma, while the study by Porterfield et al. (2017) aimed at increased capacity for quality improvement among public health practitioners. Some studies explicitly called for a shift in practice based on research findings. Kremer et al. (2017) used workshops and a knowledge-brokering team to increase the use of evidence in policy briefs. Similarly, Oronje et al. (2019) used training and mentoring programs to increase evidence use in health policymaking.

The third dimension, *scope of coverage*, refers to the number of participants. When the KT targeted specific actors or groups of actors, such as for participation in workshops or educational programs, it was classified as narrow. This applied to most of the studies in our sample (see Table 2). The scope of coverage was classified as broad when participation was relatively unrestricted, for example when using open-access Internet platforms as a means for KT. Four of the studies were classified as having broad scope of coverage.

At first glance, the fourth dimension—*orientation*—seems comparably straightforward but offers substantial room for interpretation. According to Spraggon and Bodolica (2012), people-based studies involve personal, face-to-face interactions. Typical examples include focus group discussions, workshops, and interviews. However, as these activities also can take place using distance-bridging technology, seven of the studies are classified as mixed forms, while 13 are exclusively people-oriented. Only three studies fall into the technology-oriented category. All of them (Arevalo et al. 2020; McCart et al. 2020; Porterfield et al. 2017) specifically aim at evaluating communication through an online platform. This also apply to the two technology-oriented studies included in the literature review by Campbell and Moore (2018). We can see an increase in technology-oriented studies over time.

In the addition to the dimensions, directionality is a key element in the KT process. This was not emphasized by Spraggon and Bodolica (2012) nor in most of the studies in our sample. However, the terms used offer indirect guidance, such as *knowledge transfer*, *senders*, and *receivers*. Those studies in our sample that focused on knowledge *exchange* presented a different approach (Aelbrecht and Stevens 2015; Hakkarainen et al. 2020; Hurley et al. 2017; Janousek and Blair 2018). One example is Aelbrecht and Stevens (2015), who found that informal talks stimulated two-way knowledge exchange between urban designers and researchers. “The fact that these talks were focused only on applied knowledge facilitated this. Designers were able to express easily their opinions and share their knowledge and experience” (Aelbrecht and Stevens 2015, p. 312). Packer et al. (2019, p. 81) even saw two-way communication as a requirement for effective KT in their field (“[e]ffective extension [of soil information] requires two-way communication”). Next, we explore the effects from KT processes in closer detail.

### Effects

From the theoretical background, we can primarily expect to find altered knowledge **(K)** and/or action **(A)** in studies addressing effects from KT processes. Our literature review supports this, but we also identified a third category: altered beliefs **(B)**.

Table 3 shows that altered knowledge was the most common effect explored. Most studies in our sample referred to perceived or self-assessed knowledge change after KT processes, while a few studies attempted to measure more fact-based alterations. The studies by Hains et al. (2019), McCart et al. (2020) and Oronje et al. (2019) used educational programs to increase both knowledge and beliefs or skills. Hains et al. (2019) used surveys to measure knowledge and confidence about managing patients with difficult-to-treat asthma. McCart et al. (2020) used surveys to measure effects on knowledge and self-efficacy in relation to traumatic brain injury. Oronje et al. (2019) applied surveys and interviews to investigate the effects on knowledge and skills from training and mentoring programs in health sector policymaking. The educational programs in these three studies were found to be effective. Besides increased knowledge and self-efficacy, McCart et al. (2020) noted advantages in terms of time and cost (e.g., online delivery reduced the time and costs in relation to travel). McCart et al. (2020) and Hains et al. (2019) called for further studies on more long-term changes, while Oronje et al. (2019) highlighted the importance of political commitment and institutionalization of evidence use for successful training and mentorship programs (see *Lessons from previous studies* below).

**Table 3:** Overview of reported effects from different forms of knowledge communication

Publication	Form of communication	Effects in focus	Identified effects (B: Beliefs, K: Knowledge, A: Action)	Explicit before-and-after measurement
Aelbrecht and Stevens (2015)	Presentations and informal talks	Altered knowledge on urban design and analytical capabilities	Increased knowledge **(K)**	No
Andrews et al. 2020	Storytelling and action-learning events	Work satisfaction and improved quality of life for the elderly and their carers	Increased work satisfaction **(B)** and improvements of quality of life **(A)**	No
Annemans and Heylighen (2021)	Workshops, presentations, and documents	Altered knowledge on healthcare buildings and patient experience of building design	Increased knowledge **(K)**, increased attentiveness of patient experiences **(B)** and improved communication **(A)**	No
Arevalo et al. (2020)	Storytelling	Perceived usefulness of research-based storylines	Increased knowledge of context. **(K)** Most participants considered the storylines to be useful.	No
Bruce and O’Callaghan (2016)	Discussions	Altered knowledge on policy relevant areas	Increased knowledge in some, but not all, cases **(K)**	No
Dowson (2019)	Action learning, telephone coaching	Altered knowledge and confidence (e.g., communication skills)	Self-assessed improvements of knowledge **(K)** and confidence **(B)**	Yes
Hains et al. (2019)	Educational data program	Altered knowledge and confidence on medical treatment	Increased knowledge **(K)** and confidence **(B)**	Yes
Hakkarainen et al. (2020)	Workshops	Science-society relationships, knowledge, and perceptions	Increased knowledge **(K)** and improved science-society relationships **(A)**	Yes
Hall et al. (2019)	Workshops	Application of learning about research methods	Developed library and information services, research dissemination, networking, and research collaborations **(A)**	No
Hambidge et al. 2019	Learning platform	Public engagement and knowledge of international and local issues in higher education	Improved knowledge **(K)**	No
Hurley et al. (2017)	Workshop	Improved research usability in practice	Moderately improved usability of research **(K)**	No
Janousek and Blair (2018)	Group discussion, deliberation	Improved theory-practice exchange	*	No
Kremer et al. (2017)	Workshops, writing assistance	Evidence-informed policy-making skills and, use of evidence in policy briefs **(A)**	Evidence-informed policy-making skills increased **(K)**	Yes
Lee et al. (2018)	Workshops	Perceptions of knowledge, confidence to assess persons with memory problems application of learning in clinical practice	Increased confidence **(B)** and improved ability **(K)**. Most respondents applied their learning in practice. **(A)**	No
Lundmark et al. (2019)	Lectures, deliberative group discussions	Self-assessed knowledge and policy beliefs	Self-assessed knowledge remained after 3-4 months **(K)** Effects on policy beliefs largely disappeared **(B)**	Yes
Lundmark et al. (2017)	Lectures, deliberative group discussions	Self-assessed knowledge and policy beliefs	Lectures were more effective than group deliberation to transfer knowledge **(K)** and to alter policy beliefs **(B)**	Yes
McCart et al. (2020)	Online training modules	Altered knowledge and self-efficacy	Knowledge increase (**K**) and increased self-efficacy regarding knowledge application **(B)**	Yes
Oronje et al. (2019)	Training, mentoring programs, discussion forums	Altered knowledge and skills	Improved knowledge and skills on how to use evidence in decision-making **(K)**	Yes
Packer et al. (2019)	Workshops	Self-assessed knowledge	Perceived knowledge increase **(K)**	No
Porterfield et al. (2017)	Online communication platform	Use of information and quality improvement capacity	Increased quality improvement capacity **(K)** and use of the information at work **(A)**	No
Rajaeian et al. (2018)	Collaborative research, informal communication, consultancy, presentations at seminars, publications	Awareness of implementation	Personal communication was more effective than publications. Researchers’ motivation had a positive impact **(K)**	No
Sass et al. (2019)	Educational program with practice-based learning	Altered knowledge, attitudes, and behavioral change	Increased knowledge **(K)** and communication, altered practices **(A)** and improved service satisfaction **(B)**	No
Ugolini et al. (2015)	Publications, Internet, manuals, e-learning, technical meetings, seminars, and conferences	Factors that can increase KT effectiveness (e.g., capacity for innovation, collaborations, language)	*	No
Winarto et al. (2017)	Science field-shops	Altered knowledge and habits	Improved knowledge **(K)** altered beliefs **(B)** and application of sustainable agriculture **(A)**	No
Wright and Wright (2017)	Workshops, online resource hub	Altered beliefs	Altered beliefs **(B)** (the participants became more reflexive and critically conscious)	No

Classification notes **(A, B or C)** are included when there are differences between explored and identified effects. The review articles are excluded from the table as their content in this regard is not possible to classify. Two studies (marked with *) are also unclassified, in these cases due to focus on KT design, rather than effect outcomes (se methods)

Studies on altered knowledge also relatively often explored altered beliefs (11 of the studies; see Table 3). For instance, Lee et al. (2018) used workshops to introduce a clinical reasoning approach that both resulted in improved ability of primary care clinicians to assess persons with memory problems and increased confidence to do so. Two studies (Lundmark et al. 2017, 2019) focused primarily on exploring shifts in policy beliefs from KT processes. Lundmark et al. (2017, p. 847) noted that both deliberative group discussions and lectures on genetic biodiversity in the Baltic Sea and management options “significantly changed [the managers’] view concerning the extent to which they consider genetic biodiversity of the Baltic Sea to be under threat after compared to before the knowledge communication”. The identified differences between the two forms of communication were generally small, but the authors found statistically significant alterations in beliefs in the groups with lecture communication, despite theoretically founded expectations that group discussion would have a stronger impact on participants’ beliefs. Andrews et al. (2020) provided such a link between dialogical group discussions and altered beliefs: “A dialogical approach to storytelling helps to build a common language and vision” (Andrews et al. 2020, p. 611).

Knowledge attained or created during KT processes is commonly anticipated to result in action (De Luca and Rubio 2019; Kuiken and van der Sijde 2011). Theoretically, altered knowledge and beliefs precede action (Fishbein and Ajzen 2009; Weible 2005). In the empirical studies explored herein, action is examined in about a third of the cases. Nine of the studies (Andrews et al. 2020; Annemans and Heylighen 2021; Hakkarainen et al. 2020; Hall et al. 2019; Kremer et al. 2017; Lee et al. 2018; Porterfield et al. 2017; Sass et al. 2019; Winarto et al. 2017) examined how knowledge from KT processes was put to use, predominantly through various behavioral changes among participants. For instance, 58.2% of the public health practitioners participating on the online communication platforms studied by Porterfield et al. (2017, p. 144) reported that they had used the information primarily as a tool for quality improvement. Winarto et al. (2017) identified the internalization of new habits among farmers taking part in monthly science “field-shops” together with scientists. “The scientists improved the farmers’ new habits of measuring daily rainfall and taking notes of their agroecosystem observation over time” (Winarto et al. 2017, p. 74). Hakkarainen et al. (2020) detected a link between altered knowledge and action when interacting with fishery managers and local stakeholders in Zanzibar. Among the immaterial benefits from community-level knowledge exchanges, they highlighted conceptual use of research: “Such enlightenment included for example seeing new possibilities in fish trading” (Hakkarainen et al. 2020, p. 292). Thus, conceptual use of research can cause behavior changes in the future.

Action was much more prominent in the literature review by Campbell and Moore (2018) than in our study. Eleven of 14 articles included in the literature review by Campbell and Moore reported action resulting from KT. However, this result is likely to be explained by their selection criteria, which exclusively target empirical studies on how to increase the use of research in population health policies and programs.^1^ Five of the studies identified information sharing as the main activity from KT processes. One of the studies (Kothari et al. 2014, p. 5) reported that conceptual use was more common than “instrumental use” (e.g., update of clinical protocols). Our findings are in line with these.

Five studies did not fit our three effects categories (altered knowledge, beliefs, or action). Arevalo et al. (2020) explored perceived usefulness of research-based storylines, while Hurley et al. (2017) studied usability of research among stakeholder groups. Ugolini et al. (2015) examined factors that can increase KT effectiveness. Janousek and Blair (2018) carried out group discussions among practitioners to inquire how exchange from theory to practice can be improved. Finally, Gervais et al. (2016) examined how to evaluate KT strategies.

As before-and-after measurements can be seen as a criterion of reliability, we manually scanned the articles and found eight studies that explicit used this method when exploring possible impacts from KT processes (Dowson 2019; Hains et al. 2019; Hakkarainen et al. 2020; Kremer et al. 2017; Lundmark et al. 2017, 2019; McCart et al. 2020; Oronje et al. 2019), using surveys and/or interviews to capture possible alterations. All these studies identified changes in knowledge as the result of KT processes, five identified alterations in both knowledge and beliefs, and one (Hakkarainen et al. 2020) detected change in knowledge and action. The forms of KT used varied greatly, including lectures, data programs, coaching/mentoring, action learning, workshops, group deliberation and other discussion forums (see Table 3). Three of these studies compared the effects of two or more forms of KT (Hakkarainen et al. 2020; Lundmark et al. 2017, 2019). Hakkarainen et al. (2020) used two types of workshops to explore effects on knowledge, perceptions, and science–society relationships in small-scale fisheries. They found that both community dialog and science outreach resulted in increased knowledge among managers, fishers, and traders. Lundmark et al. (2017, 2019) compared the effectiveness of two forms of KT and showed that, although both lectures and deliberative group discussions had a positive impact on managers’ self-assessed knowledge on genetic biodiversity, lectures were more effective at transferring knowledge and altering policy beliefs. As the effects disappeared within a few months, the authors concluded that continuity is more important than the form of communication.

The main take-away from our study is that it is hard to find indications regarding which form of KT works the best to promote knowledge or stimulate action. Put bluntly, effects seem possible to identify regardless of form of KT. However, few of the study in our sample attempted to study effects in a systematic way. Those that did generally found small or volatile results. Nevertheless, there are plenty of lessons to be learned from previous studies on how to bridge the gap between science and practice.

### Lessons from Previous Studies

Relevance for the target group is a recurrent theme in the studies we explored. Researchers should aim for a good match between the selected content for KT and the interests or needs of the participants. Andrews et al. (2020), who carried out storytelling and action-learning events for social care and health practitioners, managers, older people, and their next of kin, stated that research evidence must be presented in a way that attendees find meaningful (see also Arevalo et al. 2020; Janousek and Blair 2018). Similarly, Aelbrecht and Stevens (2015), who explored urban design practice, emphasized that communicated research needs to acknowledge and adapt to designers’ way of thinking; otherwise, research will not be perceived as useful. The researchers can have varying prior knowledge of the target group. Packer et al. (2019, p. 78) envisaged surveys prior to the KT as follows: “demand by farmers, agribusiness and community for useful soils information and extension can only be determined by asking them”. Relevance is not only important for the target group, researchers also benefit from relevance, as it gives access to local knowledge (Hakkarainen et al. 2020; Janousek and Blair 2018). Hurley et al. (2017, p. 520) went so far as to claim that “effective exchange and collaboration relies on concerted effort to align researcher and practitioner needs”.

Several studies addressed how to build trust through the communication process. Aelbrecht and Stevens (2015), Packer et al. (2019), and Bruce and O’Callaghan (2016) advocated two-way communication between experts and practitioners. Packer et al. (2019) and Ugolini et al. (2015) recommended practical and hands-on activities, when applicable, to involve and engage participants. Aelbrecht and Stevens (2015, p. 315) added that recurrent interaction, such as regular presence at the workplace, can also stimulate “trust and interest, and… a common language and points of reference”. Recurrent interaction was integral to the study by Bruce and O’Callaghan (2016), who explored the KT of academics during temporarily placements in policy environments. They concluded that a shared location does not guarantee effective knowledge exchange. Besides practical aspects such as pressure from other tasks (e.g., for researchers to continue publishing during their stay), they highlighted a need for further research on the effects of social relations in KT processes.

We recall from our theory section that the selected forms of communication should match the characteristics of the knowledge (Spraggon and Bodolica 2012). However, few studies in our sample addressed such a match. When analyzing end-of-life care through action learning, Dowson (2019) found that personal and professional development was enabled through coaching within an action learning approach, which offered reflection and “a safe place to face anxieties of real-world problem solving” (Dowson 2019, p. 7). In another study from the health sector, Andrews et al. (2020 p. 603), recommended research-based storytelling, as it combines intellectual and emotional components with the capacity to engage “both the minds and hearts of participants”.

Capacity to engage brings us to the researcher’s role in the KT process. Most of the studies focused on the researcher as a knowledge provider, stressing presenter characteristics such as competence and communication skills (Hurley et al. 2017; Kremer et al. 2017; Lundmark et al. 2017, 2019). As Packer et al. (2019, p. 81) put it, “[p]resenter knowledge of farming practices… significantly enhanced audience engagement”. Studies using workshops or other forms of dialog also envisioned the researcher as a dialog facilitator, building trust in the communication (Andrews et al. 2020; Hakkarainen et al. 2020; Hurley et al. 2017).^2^ A study by Campbell and Moore (2018, p. 5) noted that facilitation skills are particularly important when using technology-oriented forms of communication that “do not convey nonverbal cues”. Kremer et al. (2017) offered insights into the level of engagement of the researcher, suggesting that knowledge receptivity and knowledge use benefited from engaged and intense facilitation. Hakkarainen et al. (2020) added yet another role of the researcher when interacting with practitioners. In their study of community-level knowledge exchanges in small-scale fisheries, the researchers undertook the roles of brokers, mediating different views between the managers and fishers. Here, the “researchers found themselves stretched beyond their expected roles of knowledge providers” (Hakkarainen 2020, p. 292).

The lessons learned regarding characteristics related to the attendees primarily concerned the design of the KT and the content itself. Packer et al. (2019) suggested that diversified groups foster learning and mutual understanding. Diversified groups can also present challenges for dialog. To overcome different backgrounds, Ugolini et al. (2015) called for a common language. Arevalo et al. (2020) recommended making the material as accessible as possible, preferably including suggestions on management applications. Lundmark et al. (2017) proposed that participants can benefit from obtaining information prior to group discussions, particularly when deliberating on unfamiliar topics. Pre-workshop surveys can be used to inquire into participants’ familiarity with the topics or their needs and interests as in the study by Packer et al. (2019).

Time and other resources can facilitate (or hinder) the KT process. In this regard, however, we see a need to distinguish between different forms of KT. Some KT processes have a short time span while others (e.g., collaborative research) require considerable investments, in terms of both time and funding. Lack of time occasionally hindered practitioners from participating in KT processes in our study (Aelbrecht and Stevens 2015; Lundmark et al. 2017, 2019; Oronje et al. 2019). Kremer et al. (2017) and Bruce and O’Callaghan (2016) identified the lack of organizational support as a substantial barrier to knowledge exchange between science and practice. Hakkarainen et al. (2020) discussed previous experiences as a possible obstacle to interaction. If the participants have experienced a lack of feedback or improvements from earlier research encounters, this can obstruct new efforts and further emphasize the need to build trust in science–practitioner relationships.

Rajaeian et al. (2018) highlighted the different drivers in research and practice, and recommended a reform or the “the academic promotion system” as it does not encourage researchers to engage in practically oriented research (Rajaeian et al. 2018, p. 45). This recommendation was supported by their survey findings on extrinsic motivation, suggesting that “motivation to achieve *research publication* was negatively associated with *effective knowledge transfer*” (italics in original). Thus, impact-minded researchers were more successful in KT than publication-minded ones.

## Discussion

Our literature review shows that researchers who engage in KT to bridge the gap between science and practice have a lot to consider. Identifying the target group is rarely a problem, as researchers tend to be familiar with the empirical context to which they wish to contribute. More effort is needed to encourage active participation and, occasionally, to make the practitioners willing to participate in the first place. To encourage participation, many of the studies in our sample highlight the importance of presenting KT in a way that clearly shows its relevance. Relevance is closely linked to trust, as it signals concern for the participants and contributes to successful interaction. Trust seems to be particularly important in people-oriented studies following the typology by Spraggon and Bodolica (2012). Andrews et al. (2020, p. 612) go so far as to claim that storytelling *requires* a respectful culture that enables and encourages participants to share their experiences. Even if trust can be more important for some forms of KT, it seems reasonable to conclude that all forms benefit from trust and a respectful culture.

The main effect studied was increased knowledge (Table 3), but it was not always possible to tell whether the studies had inquired into actual or self-assessed knowledge change among the participants. This is a point worth considering in future research. We also found examples of altered skills, such as analytical capabilities (Aelbrecht and Stevens 2015; Kremer et al. 2017; Oronje et al. 2019) and altered beliefs, such as increased confidence in a work-related task (Dowson 2019; Hains et al. 2019; Lee et al. 2018). Action-oriented effects were explored in about a third of the studies. Several of these reported altered work practices, such as improved communication (Annemans and Heylighen 2021; Porterfield et al. 2017), research collaborations (Hall et al. 2019) and improved relationships between science and society (Hakkarainen et al. 2020).

Action seemed more difficult to measure than alterations in knowledge. Some actions, such as altered communication, may manifest relatively quickly, while others take considerable time. For instance, Aelbrecht and Stevens (2015, p. 314) noted that it can take many years for “built outcomes… to materialise”, as in the case of urban design. Lengthier processes call for long-term research projects, repeated interactions, and long-term commitment from practitioners (Winarto et al. 2017). A long study period also makes it more difficult to identify what can be attributed to the KT intervention and what is linked to other factors, such as organizational context. The articles in this study did not attempt to identify contextual effects on KT, though it was touched upon, for example, in the study by Bruce and O’Callaghan (2016), who argued that “the efficacy of any attempt to inform and influence policy is partly determined by the contextual conditions in which the influencing takes place”. The importance of context was most explicitly outlined in the literature review by Gervais et al. (2016, 65), arguing that “KT strategies are always undertaken in contexts that can affect their implementation and the achievement of their intended effects”.

Regardless of timespan, explicit and systematic evaluations of possible effects from KT are rare. Of the 27 articles explored herein, only eight had a design involving before and after measurements. While we find this proportion to be low, it is noticeable higher than in the literature review by Campbell and Moore (2018), where only two of 14 articles conducted pre- and post-tests to measure effects from KT. It should be noted that the study by Campbell and Moore did not focus on effects from KT processes but, rather, on interventions to promote research use in population health policies and programs. Additionally, our timespan extends to 2020, while the studies explored by Campbell and Moore are from 2011 to 2015. All but two of the studies in our sample were published between 2016 and 2020 (Table 1), suggesting that research on effects from KT have become more common in recent years. Even if the proportion of studies attempting to evaluate the effects of KT has increased over time, we agree with Campbell and Moore (2018) that more research measuring the effects from KT is needed.

In line with by Bhattacharyya et al. (2011), we also call for efforts to compare the effects from different forms of KT. Only four of the studies in our sample compared the effects of two or more forms of KT (Hakkarainen et al. 2020; Lundmark et al. 2017, 2019; Rajaeian et al. 2018), suggesting small differences between the pursued forms of KT. The presence of small changes following a KT process does not imply that the changes are negligible, as small alterations can also have a profound impact on the lives of individuals, as shown in the studies on care of the elderly (Andrews et al. 2020; Lee et al. 2018; Sass et al. 2019). Other studies in our sample point to broader implications of KT. For instance, the altered farming practices studied by Winarto et al. (2017) had positive effects on both human welfare and ecosystem management. Implications of this kind are common in the NRM field, as exemplified by Lundmark et al. (2017, 2019), who explored increased knowledge and awareness of the crucial role of genetic biodiversity among conservation managers, which, in turn, can influence their management choices.

The results of this study can have both theoretical and empirical implications for the NRM field. First, we noted that few of the studies in our sample met Spraggon and Bodolica’s (2012) criterion of matching the selected forms of communication with the characteristics of the knowledge. One aspect we conceptualized in the theory section that could facilitate such a match is context-relevant problem-solving, grounded in the practitioners’ real-life problems (also highlighted by Tedim et al. 2021). Further theoretical exploration and related empirical applications to identify which forms that are particularly useful in the NRM field would provide valuable insights. Second, the role of the researcher as a broker, mediating different views among participants, is highlighted theoretically and also found in our results. This can enrich the often-conflictual NRM field where “wicked problems” (i.e., problems rooted in fundamental differences, socially, politically or culturally, cf. Duit and Löf 2018) can hamper successful KT.

The primary constraint of this study pertains to the limited data. We tested several search strings to identify relevant articles. The selected search string covered both one-way and two-way forms of communications, but we nevertheless recognize a slight overweight in favor of the former, exemplified with knowledge *transfer* and *dissemination*. By including the terms ”knowledge communication” and ”knowledge *exchange*” we opened for two-way communications, but there clearly are other terms that can be tested in further research, for instance, knowledge sharing.

A related concern is the timespan. While our study did not explicitly delve into alterations in how KT was conceptualized and measured over time, it is clear that this topic has attracted considerably more research attention in recent years. We also find that the number of studies applying technology-oriented technics (e.g., online platforms) have become considerably more common. This trend is also reflected in the theoretical discourse surrounding KT. Technology-oriented forms of KT were introduced in the theoretical framework by Spraggon and Bodolica (2012) and emphasized even more in contemporary references particularly following the pandemic (e.g., De Luca and Rubio 2019; Fehl et al. 2022; Saide and Sheng 2021). We see a need for more updated empirical studies of effects from online KT processes.

## Conclusions

At the outset of this study, our goal was to explore what forms of KT work best to bridge the knowledge gap between science and practice. We find that few studies systematically measured effects, and even fewer compared more than one form of KT, despite the fact that most studies in our sample used more than one form of intervention. Even though our literature review does not provide a clear-cut answer, some interesting patterns can be identified, lessons learned, and paths for further research suggested. Most lessons identified are of a general character, thus applicable across different disciplines and contexts.

Methodological aspects are key when attempting to determine which forms of KT work best, for example, to increase knowledge or to change behavior. Few studies explicitly attempted to measure effects (eight of the 27 studies used before and after measurements) and even fewer explored whether the alterations, which tended to be small, persisted over time (cf. Gervais et al. 2016; Lundmark et al. 2019). Since most of the studies undertook short-term communications, minor alterations from KT are not surprising. More interesting, perhaps, is the result that most forms of KT had an effect, possibly downgrading the high importance given to the selection of form of KT in the theoretical framework by Spraggon and Bodolica (2012).

One of our research questions aimed to identify which form of communication that is most efficient. Only four of the studies in our sample compared effects from different forms of KT—and found small differences. Given that we could not find any studies with substantial differences between different forms of KT, we suggest that customization (to ensure relevance) and repeated contacts (to consolidate the learning) matter more than the form of KT.

Expertise and communication skills, highlighted in theorizing on KT (e.g., Kuiken and van der Sijde 2011; Szulanski 1996), are important but not enough for researchers who wish to interact in KT with practitioners. In addition to expertise in the topic of communication and presenting skills, researchers engaging in KT processes with practitioners also need to be able to design a KT process that enables and encourages active participation. This includes ensuring that the study is relevant (e.g., by customizing the message to match the participants’ needs or interests). To build trust, the researcher may also need to act as a broker, mediating different views among the participants. This suggests a careful composition of the research team, reinforced by professional brokers when possible. Also, the mindset of the researcher matters. Rajaeian et al. (2018) found that impact-minded researchers are more likely to engage in science–practice interaction than are researchers focusing on academic publishing. This implies that the present reward system in academia can function as a disabler of science–practice interaction.

It is not possible to draw general conclusions based on the limited data, but it seems that the selected form of communication is of lesser importance than the frequency of the interaction. As many forms are likely to have an impact, if well-constructed and implemented, more effort can be put into adjusting the study to the needs of the participants and provide continuity or repeated contacts (cf. Winarto et al. 2017). Klütsch and Laikre (2021) argued along similar lines when recommending platforms for continuous knowledge exchange on conservation genetics between scientists and managers.

This literature review suggests that the responsibility for successful KT rests with the researcher wishing to engage in science–practice interaction. However, there are also organizational factors that need to be addressed. Most notable in this study is the reward system that favors scientific publication. If the reward system should change, we may see more researchers attempting to bridge the knowledge gap between science and practice.

## Data Availability

This literature review is based on data from peer-reviewed published articles in the academic databases Scopus and Web of Science.

## Footnotes

1 Three studies included in the literature review by Campbell and Moore (2018) were within the same time span as our study (the remaining 11 are from 2011–2014). As their keywords did not match our search string (using the terms “knowledge translation” or “mobilization”), we did not identify them in our search.

2 “Presenter openness and providing answers further added to attendee trust and confidence” (Packer et al. 2019, p. 81).
